# Effect of Proton Pump Inhibitors on Colorectal Cancer

**DOI:** 10.3390/ijms21113877

**Published:** 2020-05-29

**Authors:** Takamitsu Sasaki, Shiori Mori, Shingo Kishi, Rina Fujiwara-Tani, Hitoshi Ohmori, Yukiko Nishiguchi, Yudai Hojo, Isao Kawahara, Chie Nakashima, Kiyomu Fujii, Yi Luo, Hiroki Kuniyasu

**Affiliations:** 1 Department of Molecular Pathology, Nara Medical University, 840 Shijo-cho, Kashihara 634-8521, Japan; takamitu@fc4.so-net.ne.jp (T.S.); shi.m.0310@i.softbank.jp (S.M.); nmu6429@yahoo.co.jp (S.K.); rina_fuji_comma@i.softbank.jp (R.F.-T.); brahmus73@hotmail.com (H.O.); yukko10219102@yahoo.co.jp (Y.N.); yudaihojo@outlook.com (Y.H.); isao_kawahara@a011.broada.jp (I.K.); c-nakashima@naramed-u.ac.jp (C.N.); toto1999-dreamtheater2006-sms@nifty.com (K.F.); 2 Key Laboratory of Neuroregeneration of Jiangsu and Ministry of Education, Co-Innovation Center of Neuroregeneration, Nantong University, Nantong 226001, China

**Keywords:** proton pump inhibitor, pH, gastrin, *Clostridium perfringens*, YAP

## Abstract

Proton pump inhibitors (PPIs) are administered commonly to aged people; however, their effect on colorectal cancer (CRC) has still not been fully elucidated. Here, we examined the effect of PPIs and consequent alkalization on CRC cells. PPI administration alkalized the fecal pH and increased serum gastrin concentration. PPI and pH8 treatment (alkalization) of CMT93 mouse colon cancer cells inhibited cell growth and invasion, increased oxidative stress and apoptosis, and decreased mitochondrial volume and protein levels of cyclin D1 and phosphorylated extracellular signal-regulated kinase (pERK) 1/2. In contrast, gastrin treatment enhanced growth and invasion, decreased oxidative stress and apoptosis, and increased mitochondrial volume and cyclin D1 and pERK1/2 levels. Concurrent treatment with a PPI, pH8, and gastrin increased aldehyde dehydrogenase activity and also enhanced liver metastasis in the BALB/c strain of mice. PPI administration was associated with *Clostridium* *perfringens* enterotoxin (CPE) in CRC lesions. CPE treatment activated yes-associated protein (YAP) signals to enhance proliferation and stemness. The orthotopic colon cancer model of CMT93 cells with long-term PPI administration showed enhanced tumor growth and liver metastasis due to gastrin and YAP activation, as indicated by gastrin receptor knockdown and treatment with a YAP inhibitor. These findings suggest that PPI promotes CRC growth and metastasis by increasing gastrin concentration and YAP activation, resulting in gut flora alteration and fecal alkalization. These findings suggest that PPI use in colorectal cancer patients might create a risk of cancer promotion.

## 1. Introduction

Proton pump inhibitors (PPIs) are widely used as a first-line treatment for peptic gastrointestinal disorders [1]. However, side effects from their long-term use have also been reported [2]. We previously investigated the mechanism of PPI-induced collagen colitis in terms of direct action of PPI on colonic epithelial cells, alkalization of intestinal contents, and elevation of blood gastrin levels [3]. In contrast, the effects of PPIs on colorectal cancer (CRC) are controversial.

Several reports have shown the anti-tumoral effects of PPIs. PPIs were shown to suppress proliferation of colon cancer cell lines and carcinogenesis in a rat azoxymethane (AOM) model [4]. The long-term use of PPIs was reported not to affect colorectal adenoma development or result in histological changes [5]. PPIs suppress the development and progression of cancer by suppressing the T-LAK cell-originated protein kinase that is overexpressed in various cancers [6]. PPIs also suppress the membrane-bound ATP-binding cassette transporters and reduce drug resistance in cancer [7].

However, prolonged PPI use can cause secondary hypergastrinemia [8]. It has been reported that PPI-induced hypergastrinemia leads to neuroendocrine cell hyperplasia, rebound gastric acid secretion, and *Helicobacter pylori* activation, which increases the risk of gastric cancer [9]. Long-term use of PPIs also increases the risk of CRC [10]. In CRC, the gastrin receptor (GR) is overexpressed, and gastrin-binding capacity is increased 10-fold compared to that in normal colonic epithelium [11]. Expression of gastrin and its receptor promotes progression from colorectal adenoma to cancer [12]. In a mouse CRC model, gastrin also promoted tumor growth [13].

Long-term PPI use alters intestinal flora via hypochlorhydria and alkalization, leading to intestinal infection [14]. Many studies have reported that long-term PPI use causes intestinal infection with *Clostridium perfringens* [15,16,17]. PPIs also exacerbated *C. perfringens* enteritis in a mouse model [18]. Recently, we reported that *C. perfringens* enterotoxin (CPE) enhances cancer malignancy by impairing tight junctions [19].

In this study, to elucidate the effects of PPIs on the growth and metastasis of CRC, we examined four factors; namely, PPIs, gastrin, alkaline environment, and *C. perfringens* infection, and attempted to clarify the synergistic effects of these factors on CRC.

## 2. Results

### 2.1. Effect of PPIs on Fecal pH and Serum Gastrin

**Figure 1:**
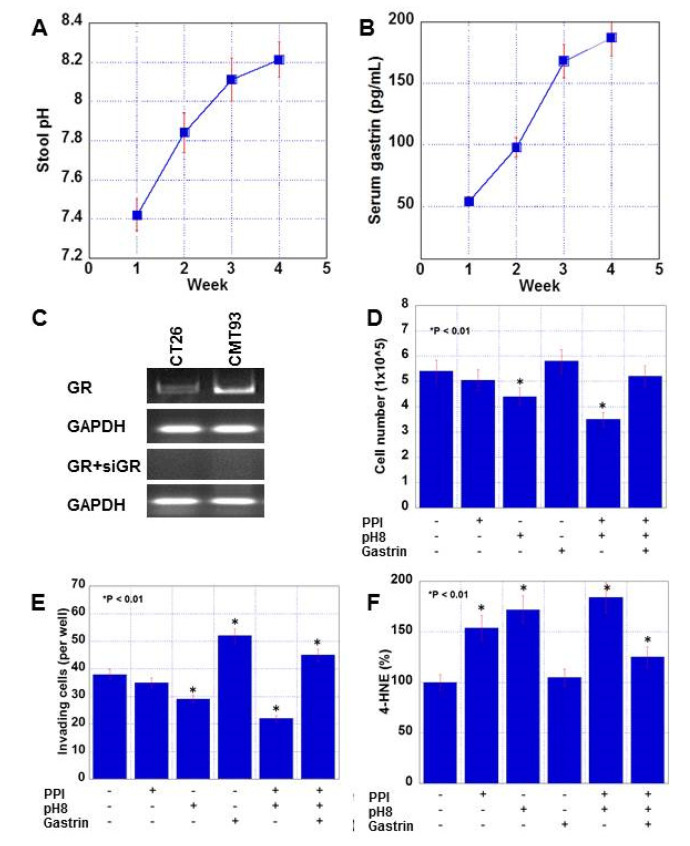
Alteration of fecal pH and serum gastrin in proton pump inhibitor (PPI)-administrated mice and effect of PPI, alkaline condition and gastrin on proliferation, invasion, and oxidative stress in CMT93 cells. (**A**,**B**) Alteration of fecal pH and serum gastrin levels in mice administered pantoprazole (25 mg/kg/day) for 4 weeks. (**C**) Expression of gastrin receptor in two mouse colon cancer cell lines and the effect of small interfering RNA (siRNA) against gastrin receptor (siGR). (**D**–**F**) Effect of pantoprazole (10 μg/mL), alkaline conditions (pH 8.0), and gastrin (150 pg/mL) for 48 h on cell proliferation (**D**), in vitro invasion (**E**), and oxidative stress (**F**). Error bar: standard error. GR, gastrin receptor.

The alterations in fecal pH and serum gastrin levels, which are the changes caused by long-term PPI administration, were examined in a mouse model. After BALB/c mice were administered PPZ (25 mg/kg body weight/day) for four weeks, stool pH and levels of serum gastrin were examined (Figure 1A,B). Stool pH was elevated to 8.21 from 7.42. Serum gastrin was increased to 191 pg/mL from 56 pg/mL.

### 2.2. Effect of PPI, pH, and Gastrin on CMT Mouse Colon Cancer Cells

**Figure 2:**
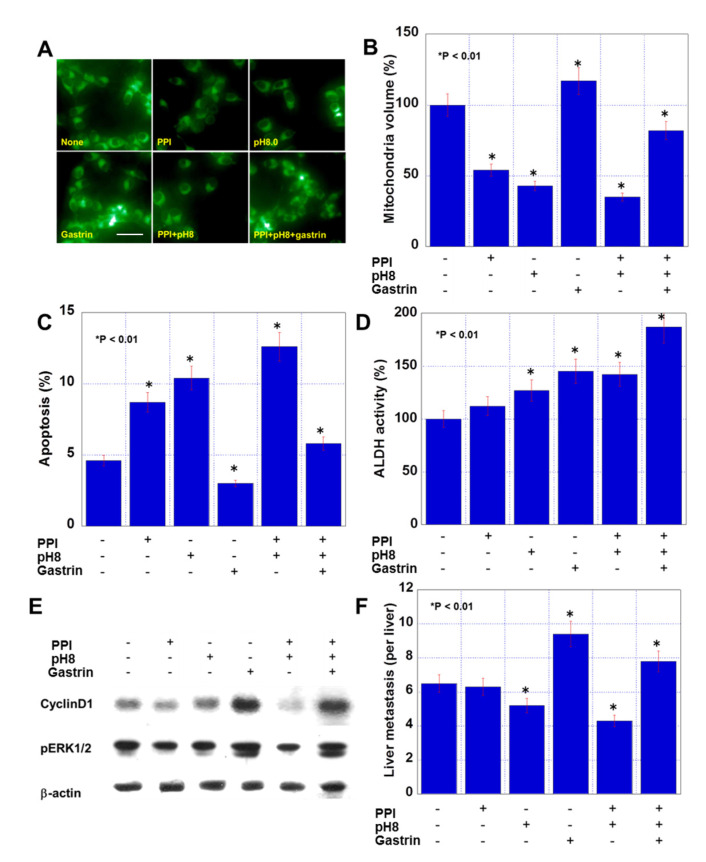
Effect of PPI, alkaline conditions and gastrin on mitochondrial volume, apoptosis, stemness, and metastability in CMT93 cells. (**A**,**B**) Mitochondrial volume assessed by MitoGreen. (**C**,**D**) Effect of pantoprazole (10 μg/mL), alkaline condition (pH 8.0), and gastrin (150 pg/mL) for 48 h on apoptosis (**C**) and stemness (**D**). (**E**) Protein levels of cyclin D1 and phosphorylated ERK1/2 (pERK1/2). β-actin served as a loading control. (**F**) Liver metastasis was examined by an intrasplenic injection of CMT93 cells treated with pantoprazole and gastrin, or exposure to alkaline condition. Scale bar: 50 μm. Error bar: standard error.

The increase in pH and the increase in gastrin level revealed by analysis of a mouse model were examined in vitro, in addition to the direct effects of the PPI. We then examined the effect of alkaline conditions (pH8 treatment) and/or gastrin (150 pg/mL) on CMT93 mouse colon cancer cells (Figure 1 and Figure 2). Both cell lines expressed cholecystokinin A/GR (Figure 1C). The PPI or gastrin alone did not affect the cell growth, whereas pH8 treatment showed growth inhibition (Figure 1D). Notably, the combination of a PPI, gastrin, and pH8 neutralized pH8-induced growth inhibition. Furthermore, the PPI alone did not affect cell invasion ability, whereas pH8 decreased invasion and gastrin enhanced invasion (Figure 1E). A combination of the three increased the number of invading cells. Both PPI and pH8 treatments showed increased 4-hydroxynonenal (HNE) levels, which reflect oxidative stress, whereas gastrin did not affect 4-HNE levels (Figure 1F). A combination of the three increased 4-HNE.

Moreover, PPI and pH8 treatment decreased the mitochondrial volume, whereas gastrin increased the volume (Figure 2A,B). A combination of the three decreased the volume. PPI and pH8 treatment increased apoptosis, whereas gastrin inhibited apoptosis (Figure 2C), and a combination of the three increased apoptosis. Stemness was evaluated by determining aldehyde dehydrogenase (ALDH) activity (Figure 2D). PPI administration did not affect ALDH activity, whereas pH8 treatment and gastrin increased ALDH activity. A combination of the three showed highest stemness. The PPI alone inhibited proliferation-associated cyclin D1 expression and extracellular signal-regulated kinase (ERK) 1/2) phosphorylation. pH8 did not affect these parameters, whereas gastrin enhanced cyclin D1 expression and ERK1/2 phosphorylation (Figure 2E). A combination of the three also showed enhanced cyclin D1 expression and ERK1/2 phosphorylation. We next examined the effect of pretreatment of the three factors on liver metastasis using a mouse model (Figure 2F). PPI-treated CMT93 cells did not show any alteration in metastasis, while the pH8 and gastrin-treated cells showed decreased and increased metastasis, respectively. A combination of the three enhanced liver metastasis.

### 2.3. Effect of CPE on CMT93 Cells

**Figure 3:**
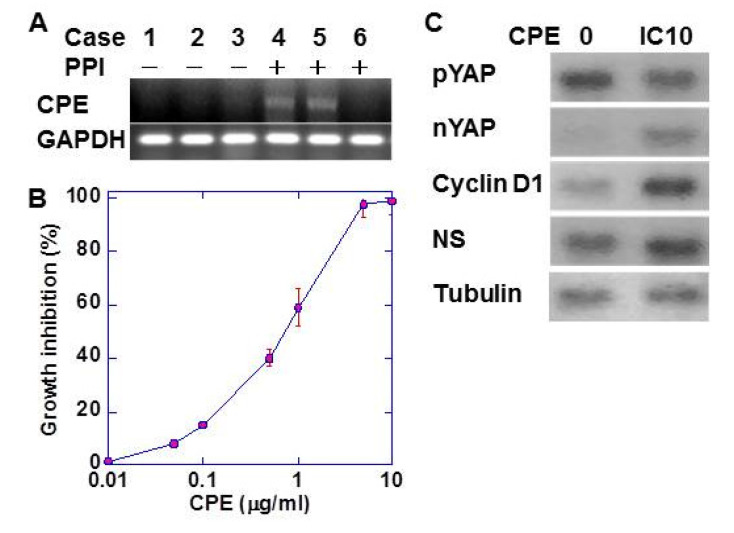
Effect of CPE in CMT93 cells. (**A**) CPE DNA was detected by PCR in CRC tissues of the patients with or without long-term (more than 6 months) PPI administration. (**B**) Growth inhibitory effect of CPE on CMT93 cells. (**C**) Effect of CPE on activation of YAP and the expression of YAP target genes. Tubulin served as loading control. Error bar: standard error. CPE, *Clostridium perfringens* enterotoxin; NS, nucleostemin; YAP, yes-associated protein; nYAP, nuclear YAP; pYAP, phosphorylated YAP.

It is known that long-term administration of PPI changes the intestinal flora, especially *Clostridium perfringens*. In cancer tissues of CRC patients, *Clostridium perfringens enterotoxin* (CPE) DNA was detected in patients undergoing long-term PPI administration (Figure 3A). We then examined the effect of low-dose CPE on cell proliferation of CMT93 cells (Figure 3B). CPE showed growth inhibition in a dose-dependent manner. CMT93 cells treated with low-dose CPE (IC_10_) showed a decrease in phosphorylated yes-associated protein (YAP) and an increase in the expression of nuclear YAP, cyclin D1 (proliferation), and nucleostemin (NS; stemness) (Figure 3C).

### 2.4. Effect of PPIs on CMT93 Mouse Tumors

**Figure 4:**
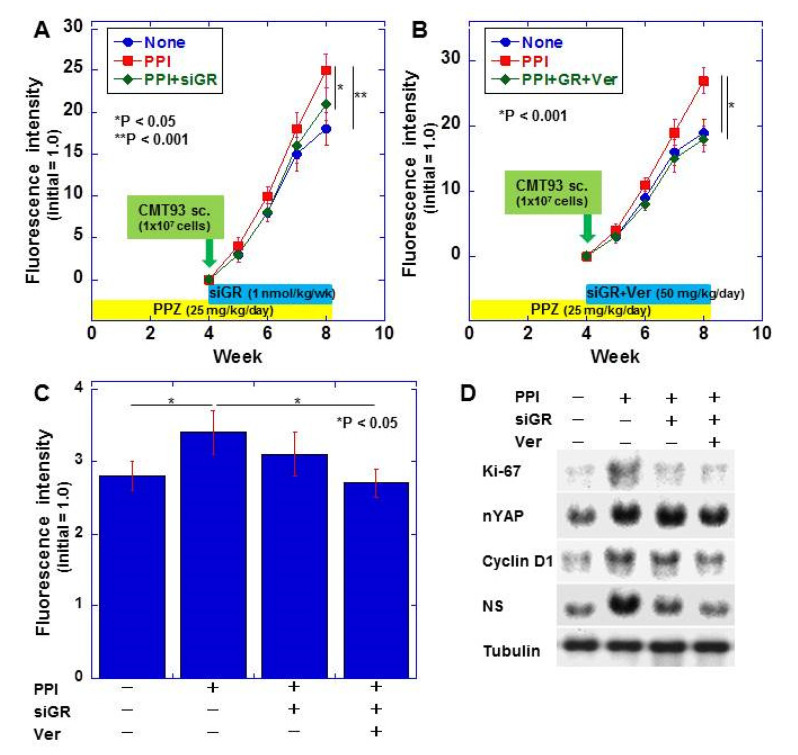
Effect of PPI on tumor growth in orthotopic cecal tumors of CMT93 cells. (**A**) Effect of PPZ and/or knockdown of gastrin receptor (siGR) on tumor growth. (**B**) Effect of PPZ and/or knockdown of gastrin receptor (siGR) plus YAP inhibition by Ver on tumor growth. (**C**) Spontaneous liver metastasis of the orthotopic cecal tumors. (**D**) Effect of CPE on activation of YAP and the expression of YAP target genes in the cecal tumors. Tubulin served as loading control. Error bar: standard error (*n* = 5 mice). PPZ, pantoprazole; siGR, siRNA against gastrin receptor; Ver, verteporfin; NS, nucleostemin; nYAP, nuclear YAP.

Finally, the effect of PPI on colorectal cancer was examined using a mouse model by inhibiting gastrin and YAP. We examined the effect of PPIs on CMT93 orthotopic cecal tumors using mice orally administered with a PPI (Figure 4). The PPI-administered mice showed more enhanced tumor growth than the untreated mice (Figure 4A). Tumor growth was partially inhibited in GR-knockdown mice treated with the PPI. In contrast, when GR-knockdown mice were simultaneously treated with verteporfin to inhibit YAP and a PPI, the tumor growth was inhibited to the same level as that in untreated mice (Figure 4B). Moreover, PPI administration increased liver metastasis of CMT93 cells (Figure 4C). In contrast, when GR-knockdown mice were treated concurrently with PPI and verteporfin, liver metastasis was inhibited to the same level as that in untreated mice. PPI treatment increased cell proliferation (Ki-67) and activated YAP and increased YAP-associated cyclin D1 and NS expression (Figure 4D). In contrast, GR knockdown and verteporfin neutralized the PPI-activated YAP signals.

## 3. Discussion

In this study, PPI-induced hypergastrinemia, alkalization of colonic content, and increased *C. perfringens* in the intestinal tract promoted colon cancer growth, stemness, and metastasis. There are various reports on the effects of gastrin on CRC. Gastrin expression is found in more than 60% of CRC and promotes proliferation and tumorigenesis [20]. Gastrin promotes DNA synthesis 1.4 times more in CRC cells compared with untreated cells [21]. In addition, systemic hypergastrinemia enhances colorectal carcinogenesis and CRC promotion [22]. In contrast, it has been reported that gastrin suppresses the early growth response gene-1/anion exchanger-2/P16/P-ERK signaling pathway to inhibit the growth of colon cancer [23]. In our study, gastrin promoted cell proliferation, its invasive potential, anti-apoptotic survival, and metastasis.

GR (cholextokinin-1 receptor) is overexpressed in CRC and is involved in cancer progression and poor prognosis [24]. The GR expression was also confirmed in the colon cancer cell line used in this study. In a mouse model using the MC26 mouse colon cancer cell line, suppressing the GR with its antagonist (proglumide) suppressed the tumor growth and prolonged mouse survival [25]. Thus, CRC cells express GR and gastrin to promote growth in an autocrine manner [12].

In colon carcinogenesis, co-expression of gastrin and its receptor occurs in the early stage in the adenoma-carcinoma sequence [12]. The mutation of the *ras* gene, which is a relatively early event in colorectal carcinogenesis [26], induces the gene expression of gastrin and its receptor [27,28]. In our mouse model, continuous administration of PPI resulted in an increase in serum gastrin levels. Knockdown of GR suppressed tumor growth and metastasis. Thus, hypergastrinemia due to PPI administration was considered to promote the development of CRC.

Reduction of gastric acid secretion by PPI results in alkalization of intestinal contents [29]. In this study, fecal pH was increased by continuous administration of PPI. It has been reported that increasing the extracellular pH results in increased intracellular pH, which is associated with decreased cell proliferation, smaller spheres, and an increase in doxorubicin uptake [30]. Our data also showed that an alkaline environment alone led to suppression of cell proliferation and invasion, and increased oxidative stress and apoptosis. In contrast, co-treatment of gastrin and PPI with alkaline conditions increased stemness and liver metastasis. However, the mechanism of the synergism is not clear. Further investigation is needed to reveal the mechanism.

It has been reported that reduction of intestinal acidity by PPI results in alteration of intestinal bacterial flora [31]. Particularly, PPI is known to increase *C. perfringens* in intestinal flora [32,33,34]. Intestinal *C. perfringens* increases 10-fold due to decreased gastric acid secretion [29]. In our analysis of CRC patients, CPE was also detected in patients receiving long-term administration of PPI. We previously reported that CPE produced by *C. perfringens* impairs CLDN4, which forms tight junctions, to promote cancer progression by activating YAP [19]. Consistent with this, in the present study, CPE activated YAP to enhance the expression of cyclin D1 and NS, which are target genes of YAP. YAP activation was also observed in cancer cells in the PPI-administered mouse model.

We examined the overall effect of PPIs on CRC using a mouse model. An orthotopic CRC model was employed to observe effects such as alkalinization of intestinal contents and changes in intestinal flora, which are also observed in human CRC. The results indicated that PPI promoted tumor growth. In contrast, knockdown of GR alone showed partial suppression; however, the combined use of GR knockdown with verteporfin, which is a YAP inhibitor, suppressed the PPI-mediated tumor growth to the level observed in the control mice. The use of a PPI also promoted liver metastasis, which could be abrogated by the combined use of GR knockdown and verteporfin.

PPIs have also been reported to inhibit the excretion of intracellular protons from breast cancer cells [35] and to induce anticancer drug sensitivity and apoptosis [36]. However, CRC differs from breast cancer in that it is affected by intestinal *C. perfringens* along with PPIs and gastrin in the blood. In our mouse orthotopic model, a combination of a PPI, gastrin, and CPE was shown to promote tumor growth and metastasis. In the future, the effect of PPIs on anticancer drug sensitivity, as observed in breast cancer cells, should be examined. Based on the results of our research, it is necessary to conduct clinical studies on the cancer-promoting effects of PPI use in colorectal cancer patients.

## 4. Materials and Methods

### 4.1. Cell Line

The CMT93 mouse rectal cell line was purchased from DS Pharma Inc. (Osaka, Japan). Cells were cultured in Dulbecco’s modified Eagle’s medium (DMEM; WAKO Purechemical Ind. Ltd., Osaka, Japan) supplemented with 10% fetal bovine serum (Sigma Chemical Co., St. Louis, MO, USA) at 37 °C in 5% CO_2_. Alkaline (pH 8.0) medium was made by adding Tris-HCl (pH 9.0, Sigma) into DMEM. Cell growth was assessed using 3-(4,5-dimethylthiazol-2-yl)-2,5-diphenyltetrazolium bromide (MTT) assay (Sigma), as previously described [37]. Human gastrin was purchased from R&D Systems Inc., (Minneapolis, MN, USA).

### 4.2. Assessment of Cell Growth and Apoptosis

The cells (1 × 10^4^ per well) were seeded in a 12-well dish. Cell growth was assessed by MTT assay or by counting the number of cells with an Autocytometer (Sysmex, Kobe, Japan) after 48 h, as described previously [37]. Apoptosis was assessed using an apoptosis/necrosis detection kit (Enzo Life Sciences, Plymouth Meeting, USA). Annexin V-EnzoGold present in the kit fluorescently labeled apoptotic cells. Apoptosis was determined by examining 2000 cells under a fluorescent microscope (Leica Microsystems, Tokyo, Japan). All the experiments were performed in triplicate.

### 4.3. Chamber Invasion Assay

A modified Boyden chamber assay was used to examine the in vitro invasion of CMT93 cancer cells [38]. Following incubation at 37 °C for 24 h, the filters were carefully removed from the inserts, stained with hematoxylin for 10 min, and mounted on microscopic slides. The number of stained cells in each insert was counted at 100× magnification. Invasion activity was quantified by calculating the average number of cells per insert well. These experiments were performed in triplicate.

### 4.4. Animals

Four-week-old male BALB/c mice were purchased from SLC Japan (Shizuoka, Japan). The animals were maintained according to institutional guidelines approved by the Committee for Animal Experimentation of Nara Medical University (Approval Number 15112, approved on 12 November 2016), in accordance with the current regulations and standards of the Japanese Ministry of Health, Labor, and Welfare.

### 4.5. Animal Models

To establish an orthotopic colon tumor model, CMT93 cancer cells (1 × 10^6^ cells in 30 mL of Hanks’ balanced salt solution) were inoculated into the cecal wall (submucosal layer) of BALB/c mice using 28G needles under inhalation anesthesia of sevoflurane (Maruishi Pharmaceutical Co. Ltd., Osaka, Japan) according to the previously published method [39,40]. The mice were observed for 4 weeks following inoculation. After 4 weeks, spontaneous liver metastasis was found.

Pantoprazole (PPZ, Sigma, 50 mg/kg) was administered every two days by oral gavage. With five mice in each group, small interfering RNA (siRNA) against GR (siGR, 500 pmol/kg body weight, twice a week, i.p, Santa Cruz Biotechnology (Santa Cruz, CA, USA)) and/or verteporfin (Ver, 50 mg/kg body weight, every two days by oral gavage, Cayman Chemical, Ann Arbor, MI, USA) were administered. A vehicle control group was run in parallel. Mice were fed with a standard CE-2 diet (CLEA Japan, Tokyo, Japan).

### 4.6. In Vivo Imaging of Tumor

CMT93 cells were labeled with VivoTrack 680 (PerkinElmer Inc., Waltham, MA, USA). A mouse was examined using the Clairvivo OPT in vivo imager (Shimazu, Kyoto, Japan) under anesthesia [41,42]. The fluorescence intensity was calculated with software equipped in the imager. The fluorescence intensity of the tumor was expressed relative to the intensity of 1 × 10^6^ cells, which was set at 1.0.

### 4.7. Stool pH

At euthanasia, intracolonic stool was collected and suspended in distilled water (2 mL), which was centrifuged (500× *g*, 5 min). The supernatant was subjected to pH measurement using a pH meter (SK-620PH, SATO Keiryoki MNG. Co., Ltd., Tokyo, Japan).

### 4.8. SiRNA

FlexiTube siRNA targeting mouse cholecystokinin A/GR was purchased from Santa Cruz Biotechnology (Santa Cruz, CA, USA). AllStars Negative Control siRNA (Qiagen) was used as a control. Mice inoculated with CMT93 cells were treated with siRNA encapsulated with liposome, which was administered intraperitoneally; iRNA (100 pmol) was encapsulated with 2 mL of cationic liposome (EL-C-01, Nippon-Oil&Fats Co., Tokyo, Japan) [43], and 200 μL of the solution was administered intraperitoneally in each mouse twice a week [44].

### 4.9. Reverse Transcription-Polymerase Chain Reaction (RT-PCR)

To assess mRNA expression, RT-PCR was performed with 0.5 µg total RNA extracted from cells using an RNeasy kit (Qiagen, Germantown, MD, USA). The primer sets for mouse cholecystokinin A/GR amplification were as follows: forward, 5′-CCA CCT ACT TCA TGG GCA CT-3′ and reverse, 5′-GTT CGC CGT CTG GTT ATT GT-3′ (NCBI Reference Sequence: BC020534.14; synthesized by Sigma Genosys, Ishikari, Japan). PCR products were electrophoresed in a 2% agarose gel and stained with ethidium bromide. *GAPDH* mRNA was also amplified for use as an internal control (GenBank Accession No. NM_001289726.1).

### 4.10. Western Blotting Analysis

Whole-cell lysates were prepared using 0.1% nonidet P-40 containing lysis buffer as previously described [38]. Cell fractions were extracted using a Cell Fractionation Kit (Abcam, Cambridge, MA, USA), according to the manufacturer’s instructions [45]. The nuclear fractions were subjected to analysis to detect nuclear YAP.

Proteins (20 μg) present in cell lysates were separated by performing sodium dodecyl sulfate-polyacrylamide gel electrophoresis on 12.5% gels and were electrotransferred onto nitrocellulose membranes for immunoblotting analysis. The membranes were incubated with primary antibodies, followed by incubation with peroxidase-conjugated IgG antibodies (MBL). Tubulin antibody (Oncogene Research Products, Cambridge, MA, USA) was used to measure the amount of protein loaded per lane. Immune complexes were visualized using the CSA system (DAKO, Carpinteria, CA, USA). Antibodies against Ki-67, YAP1, Cyclin D1 (Abcam), nucleostemin (NS), phosphorylated extracellular signal-regulated kinase 1/2 (ERK1/2, Thr202/Tyr204) (Santa Cruz) and phosphorylated YAP1 (pSer127, Biorbyt LLC., St. Louis, MO, USA) were used as primary antibodies.

### 4.11. Enzyme-Linked Immunosorbent Assay (ELISA)

Whole-cell lysates were prepared using 0.1% nonidet P-40 containing lysis buffer as previously described [38]. Mouse gastrin ELISA kit (Cusabio Technology LLC., Houston, TX, USA) and 4-hydroxynonenal (4HNE) ELISA kit (R&D Systems) were used to measure gastrin and 4-HNE levels in the cell lysate according to the manufacturers’ instructions.

### 4.12. Mitochondrial Volume

Mitochondrial volume was assessed by staining mitochondria with MitoGreen (Takara Bio Inc., Kusatsu, Japan). Cell images were captured under a microscope equipped with a camera (Leica Microsystems, Tokyo, Japan), and fluorescence was measured in the images using Photoshop Image Analyzer (Adobe Systems Inc., San Jose, CA, Japan).

### 4.13. Aldehyde Dehydrogenase (*ALDH*) Activity

ALDH activity was measured using the ALDEFLUOR kit (Veritas, Tokyo, Japan) according to the provider’s instructions. The activity was normalized to the negative control N,N-diethylaminobenzaldehyde (DEAB) [46].

### 4.14. Surgical Specimens

We reviewed the pathological diagnosis and clinical data of 6 CRC patients with or without PPI administration longer than 3 years in the Department of Molecular Pathology, Nara Medical University, Japan. As written informed consent was not obtained from the patients in this retrospective study, any identifying information was removed from the samples prior to analysis, to ensure strict privacy protection (unlinkable anonymization). All procedures were performed in accordance with the Ethical Guidelines for Human Genome/Gene Research issued by the Japanese Government and were approved by the Ethics Committee of Nara Medical University (approval number 937).

### 4.15. Bacterial DNA Amplification

Bacterial DNA detection by PCR was performed as previously described [19]. Bacterial DNA was extracted from the CRC specimens (10 thin-sliced paraffin-embedded tumor specimens, deparaffinized and hydrated) using the QIAamp DNA mini kit (Qiagen, GmbH, Hilden, Germany) according to the manufacturer’s instructions. The extracted DNA samples were stored at −20 °C. The PCR cycle comprised an initial denaturation step (94 °C for 5 min), followed by 35 cycles of denaturation (94 °C for 1 min), annealing (50 °C for 1 min), and primer extension (72 °C for 1.5 minutes). Amplified PCR products were analyzed by 1.5% agarose gel electrophoresis in Tris-Borate-EDTA) buffer. The gel was stained with 0.5 μg/mL ethidium bromide. The primer sets used for the amplification of CPE DNA were as follows: forward, 5′-TCC CCT TTC TAG ATA ACG ATT AAC AC-3′ and reverse, 5′-GTT AGC ATG CTG TTT TCT AAG TTA AAA CC-3′ [47]. Primers were synthesized by Sigma Genosys (Ishikari, Japan).

### 4.16. Statistical Analysis

Statistical significance was calculated by two-tailed Fisher’s exact, Chi-squared, and unpaired Student’s *t*-tests using InStat software (GraphPad, Los Angeles, CA, USA). Statistical significance was defined as a two-sided *p*-value of < 0.05.

## 5. Conclusions

In this study, we investigated the effect of PPI on CRC in vitro and in vivo, and showed a tumor promoting effect. Since PPI is a drug that is widely used clinically, it will be necessary to consider its effect on CRC in the future.
